# Social Support and Resilience as Mediators Between Stress and Life Satisfaction Among People With Substance Use Disorder in China

**DOI:** 10.3389/fpsyt.2018.00436

**Published:** 2018-10-16

**Authors:** Chunyu Yang, Mengfan Xia, Mengmeng Han, Ying Liang

**Affiliations:** ^1^School of Economics and Management, Changzhou Institute of Technology, Changzhou, China; ^2^School of Social and Behavioral Sciences, Nanjing University, Nanjing, China

**Keywords:** stress, social support, resilience, life satisfaction, people with substance use disorder

## Abstract

This study investigated the potential mediating roles of resilience and social support in the relationship between stress and life satisfaction. A total of 426 individuals, who have substance use disorder, from the Shifosi and Dalianshan rehabilitation facilities in China participated in the study. They were tested using the Perceived Stress Scale, Multidimensional Scale of Perceived Social Support, Connor–Davidson Resilience Scale, and Satisfaction with Life Scale. Results showed that the serial multiple mediation of social support and resilience in the relationship between stress and life satisfaction was significant. Furthermore, the findings corroborate the important roles of perceived social support and resilience in alleviating stress. Finally, we discussed ways to enhance the life satisfaction for individuals who have substance use disorder and analyzed the limitations of this study.

## Introduction

Drug abuse is an important and widespread health problem (1). Statistics from the Niaz et al. (2) indicate that approximately 29.5 million adults worldwide use illegal drugs, which account for approximately 5.3% of the global population (3). In China, the use of drugs by 2.51 million people was registered by the end of 2016 (excluding retraining, deaths, and departures after 3 years of abstinence), with an annual increase of 6.8% (4). The prevalence of drug abuse and the increasing number of people with substance use disorder impeded China's social and economic development. The direct economic loss caused by drug abuse in China amounts to several hundred billion yuan annually. In addition, extreme social behaviors, such as robbery, theft, violence, and self-inflicted injuries, caused by drug abuse have seriously endangered the healthy development of the society. Research has emphasized that people who are dependent on drugs face more pressure than other groups (5, 6). They deal with stigma among family and friends, pressures in employment and life, social integration, physical and mental dependence, loss of self-identity, financial problems, and lack of institutional assistance (7–11). The negative reinforcement processing model of addiction shows that escaping from negative emotions caused by the negative external environment is the dominant motivation for maintaining addictive behaviors (12). Studies have found that stress plays an important role in drug abuse and its persistence (13, 14). Stress may be a common factor in promoting the memory of dorsolateral dependence, which can be used as a neural mechanism to increase drug use and its relapse after stressful life events (15). Stress forces people who use drugs to relapse after they return to society. Tartaglia et al. (16) performed a regression analysis to test the relationship between cannabis use and life satisfaction. They found that life satisfaction is negatively related to substance use. Drug dependents frequently feel depressed, anxious, and even suicidal (17, 18). Therefore, considerable attention must be accorded to the pressure and health problems experienced by people with substance use disorder.

### Stress and life satisfaction

With the development of positive psychology, attention toward life satisfaction has increased in the academic literature (19–21). Life satisfaction refers to a subjective assessment of the quality of life and is considered an important component of subjective well-being (22). It is also an indicator of psychological states. Life satisfaction is a resource that includes autonomy, control beliefs, positive emotions, emotional regulation, problem-solving, adaptation, and balance throughout the life cycle (23). In contrast, perceived stress is a subjective evaluation of an aversive situation. Stress has been studied by measuring the physiological performance, the occurrence of major life events, and cognitive evaluation (24). Stress occurs when the demand for events exceeds the available resources (25). The relationship between stress and life satisfaction has been the subject of a considerable research (26–28). Numerous empirical studies have shown that stress is negatively related to life satisfaction (26, 29, 30). Stress exerts a negative effect on people over time, which results in dissatisfaction with life and other emotional reactions. The previous literature has shown that stress is associated with life satisfaction; however, the underlying mechanisms behind such a relationship remain unclear. Therefore, the present study aims to identify the potential intermediary mechanisms between stress and life satisfaction.

### Stress, social support, resilience, and life satisfaction

Social support is hypothesized as a mediator between stress and life satisfaction. Some studies have shown that individuals with extremely high stress levels rarely feel satisfied with themselves and are likely to have low social support (31, 32). These conditions are negatively related to their physical and psychological well-being (33). People with higher levels of social support have been proved to be less likely to use drugs and alcohol (34, 35). Social support is associated with better quality of life and acts as a significant indicator of the subjective well-being among people with substance abuse disorder (36). Wang et al. (37) found that the relationship between stress and life satisfaction can be mediated by support from family and friends, but not from a person's significant other. Stress is associated with life satisfaction by increasing the demand for social support. In other words, the level of social support can mediate the relationship between stress and life satisfaction.

Resilience is also hypothesized as a mediator between stress and life satisfaction. Stress has been found to be positively correlated with decreased resilience (38, 39). Long-term stress exposure undermines a person's successful adaptation to a threatening environment, which is not conducive to the development of resilience (27). Research has shown that resilience is an important psychological resource that can maintain or recover high well-being while confronting life's adversities (40–42). Resilient individuals can maintain physical and mental health by alleviating the negative consequences of difficult situations (43, 44). Resilience has been positively identified as an important source of life satisfaction. Shi et al. (45) found that resilience plays the role of a partial mediator in the relationship between stress and life satisfaction among Chinese medical students.

Considerable literature has addressed the relationship between stress and life satisfaction (46, 47). Moschion and Powdthave (48) in a longitudinal study of 1174 respondents found that a decrease in life satisfaction following the consumption of illegal/street drugs persists 6 months to a year after use. Laudet and White (49) have shown that higher life satisfaction and less stress are positively associated with high levels of social support among individuals with substance use disorder. Nikmanesh and Honakzeh (34) found that enhancing perceived social support and positive affection plays a significant role in increasing teenagers' resilience to drug abuse. Individuals with resilience were less likely to involve themselves in drug abuse (50), which is beneficial to life satisfaction (51). To our knowledge, however, no study has yet assessed whether the relationships among the three variables (i.e., stress, social support, and resilience) can simultaneously affect the life satisfaction of people with substance use disorder. The relationships among stress, social support, resilience, and life satisfaction of people with substance use disorder remain unexplored. Therefore, the potential mediating roles of social support and resilience in the relationship between stress and life satisfaction among people with substance use disorder may be critical to key decision makers when developing intervention strategies for the treatment process.

Accordingly, the present study aims to verify the mediating roles of social support and resilience in the synergic effect of the relationship between stress and life satisfaction. On the basis of the summary of the existing studies on the relationship among stress, life satisfaction (46), resilience (52), and social support (53), we hypothesized that social support and resilience act as mediators between stress and life satisfaction among people with substance use disorder in China.

**Figure 1:**
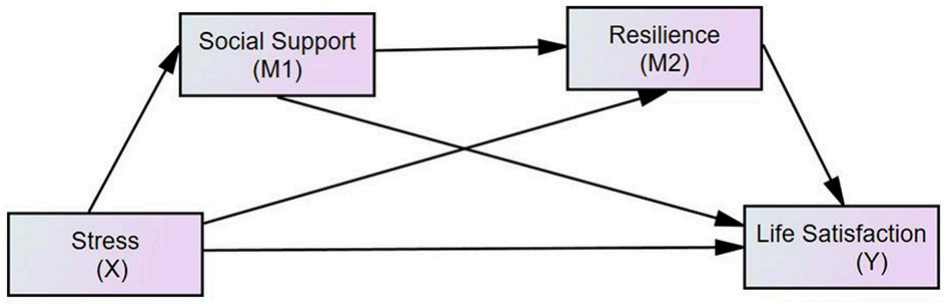
The hypothesized model concerning the relationship between stress and life satisfaction: social support and resilience as mediators.

In addition, the existing research results show that as a social resource, social support directly affects resilience (54). Given the significant influence of resilience on life satisfaction, stress is assumed to exert a considerable indirect effect on life satisfaction by mediating the effects of social support and resilience. In particular, individuals with low stress perception receive high social support. Their mental flexibility is also improved. Thus, their life satisfaction is higher than that of the individuals with high stress perception. Social support and resilience play significant intermediary roles in the relationship between stress and life satisfaction. The hypothesis model is shown in Figure 1.

## Methods

### Participants and procedure

A total of 426 people with substance use disorder from China volunteered to participate in the study without compensation. Informed consent was obtained from all the participants prior to the initiation of the investigation. The participants were asked to independently complete the questionnaire in a conference room to ensure the confidentiality of their information. The questionnaire took approximately 30 min to complete.

The 426 participants, comprising 328 males and 95 females (excluding the missing ones), were from the Shifosi and Dalianshan rehabilitation facilities in China. Their age ranged from 19 to 51 years. For the education variable, the level of education degrees was considered as a reference criterion: 1 = elementary school and below, 2 = middle school, 3 = high school, and 4 = college and above. The distributions of the “1” and “3” variables were similar, i.e., each level accounted for nearly 20% of the participants. Meanwhile, variable “2” comprised the largest proportion (*P* = 53.1%), whereas variable “4” had the smallest proportion (*P* = 7%). Considering the marital status, 31.2% (*n* = 133) of the participants were single, 33.3% (*n* = 142) were married to a living spouse, 31.5% (*n* = 134) were divorced, and 2.3% (*n* = 10) were widowed. The drug abuse analysis indicated that 59.5% (*n* = 280) of the participants were addicted to meth, 26.3% (*n* = 112) to heroin, 2.1% (*n* = 9) to marijuana, 1.2% (*n* = 5) to cocaine, 0.9% (*n* = 4) to ecstasy, 0.7% (*n* = 3) to morphine, and 1.2% (*n* = 5) to other drugs. Furthermore, 56.4% of the respondents reported an annual income below 50,000 yuan, 20.4% between 50,000 to 10,000 yuan, and 19.7% < 10,000 yuan.

### Measures

#### Stress

The Perceived Stress Scale (PSS) was administered to assess stress. It consists of 14 items, which measure the degree of stress experienced by the respondents over the past 4 weeks. PSS-14 uses a five-point Likert scale response format (from 0 = never to 4 = very often) (25). The PSS-14 score is based on a summary of all 14 projects. The Chinese version of PSS-14 achieves good levels of reliability (0.808) and validity among the Chinese population (55).

#### Social support

The Multidimensional Scale of Perceived Social Support (MSPSS) was administered to assess social support. In this scale, the participants respond to each question using a seven-point Likert scale (from 1 = very strongly disagree to 7 = very strongly agree). MSPSS focuses on three main subscales: family, friends, and significant other (56). The Chinese version of MSPSS achieves good levels of reliability and validity among the Chinese population (57, 58). The Cronbach's alpha of MSPSS in this study is 0.911, which indicates the high reliability of this scale.

#### Resilience

The Connor–Davidson Resilience Scale (CD-RISC) was administered to assess resilience. The CD-RISC comprises 25 items, which can be rated using a five-point scale (0 = not true at all, 1 = rarely true, 2 = sometimes true, 3 = often true, 4 = true nearly all the time); a high score reflects greater resilience (59). The Chinese version of CD-RISC achieves good validity and reliability among the Chinese population (60). The Cronbach's alpha of CD-RISC in this study is 0.908, which indicates the high reliability of this scale.

#### Life satisfaction

The Satisfaction with Life Scale (SWLS) was administered to assess life satisfaction. SWLS consists of five statements. Participants will indicate their degree of agreement to these statements using a seven-point Likert scale. The five statements are listed below. (1) In most ways, my life is close to my ideal. (2) The conditions of my life are excellent. (3) I am satisfied with my life. (4) So far, I have achieved the important things I want in life. (5) If I could live my life over, I would change almost nothing. The seven-point scale is as follows: 1 = strongly disagree, 2 = disagree, 3 = slightly disagree, 4 = neither agree nor disagree, 5 = slightly agree, 6 = agree, and 7 = strongly agree. The SWLS score is derived by summarizing the rating of each participant for the five statements (61). The Chinese version of SWLS achieves good validity and reliability (57, 60, 62). The Cronbach's alpha of SWLS in this study is 0.844, which indicates the high reliability of this scale.

## Results

### Preliminary analyses

We used an initial correlational analysis to test the relationships among stress, social support, resilience, and life satisfaction. The descriptive statistics included mean and standard deviation (SD), which were tested using IBM SPSS Statistics version 22.

The descriptive statistics (mean, SD, and alpha), reliability estimates (Cronbach's alpha coefficients), and correlations of all the variables are presented in Table 1. The results show significant correlations among all the variables. Stress was negatively related to social support, resilience, and life satisfaction, whereas life satisfaction was positively related to social support and resilience. These bivariate correlations support the following mediation analyses.

**Table 1:** Means, standard deviations (SD), Alpha, reliabilities and intercorrelations among study variables.

**Number**	**Measure**	**Mean**	**SD**	**Alpha**	**1**	**2**	**3**	**4**
1	Stress	41.11	5.50	0.808	1			
2	Social Support	52.86	13.61	0.911	−0.222^**^	1		
3	Resilience	77.74	16.46	0.908	−0.389^**^	0.484^**^	1	
4	Life satisfaction	16.39	6.63	0.844	−0.100^*^	0.236^**^	0.261^**^	1

*α, Cronbach's alpha*.

** *Correlation is significant at the 0.01level(2-tailed)*.

* *Correlation is significant at the 0.05level(2-tailed)*.

### Serial multiple mediation model

A serial multiple mediation model was used to test the important roles of social support and resilience in mediating the relationship between stress and life satisfaction. Compared with the traditional mediation method, a serial multiple mediation model enables researchers to simultaneously analyse two or more mediators. Furthermore, it can provide effective values for each model path and account for other model paths. In accordance with the development of multiple mediation macros presented by Preacher and Hayes (63), we calculated the standard value of the direct and indirect coefficients in the relationship between stress and life satisfaction. All the path coefficients stand for regression weights in the relationship between independent and dependent variables.

**Figure 2:**
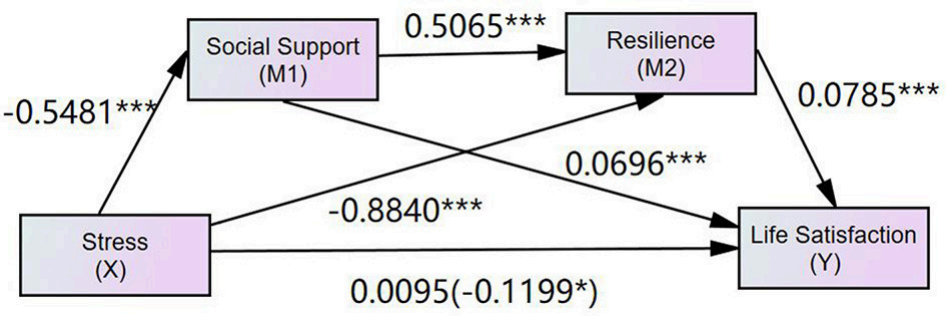
The finalized structural model (*N* = 426) in the present study. Factor loading are standardized. Support, perceived social support; SS1-SS3, three parcels of social support; SS1, family support; SS2, friend support; SS3, specialist support; LS1-LS3, three parcels of life satisfaction; PSS1-PSS3, three parcels of stress; Re1-Re2, two parcels of resilience.

As shown in Figure 2, the total effect (β = −0.1199, *p* < 0.05) from stress to life satisfaction was at a significant level (Step 1). Moreover, the direct paths from stress to social support (β = −0.5481, *p* < 0.001) and resilience (β = −0.8840, *p* < 0.001) were significant. Meanwhile, the paths from the first mediator (social support) to the second mediator (resilience) were also significant (β = 0.5065, *p* < 0.001) (Step 2). The paths from the mediators, namely, social support (β = 0.0696, *p* < 0.001) and resilience (β = 0.0785, *p* < 0.001), to life satisfaction were significant (Step 3). However, the direct path from stress to life satisfaction was insignificant (β = 0.0095, *p* > 0.05) (Step 4). Moreover, the mediating variables (social support and resilience) were observed to exert a mediating effect on the relationship between stress and life satisfaction.

The bootstrapping procedures in the SPSS PROCESS macro from the serial multiple mediation model 6 were used to test the significance of the indirect effects of stress on life satisfaction through the mediation of social support and resilience (64). Following the recommendations of Shrout and Bolger (65), we generated 10,000 samples from the original dataset (*N* = 426) via random sampling. If the 95% confidence interval (CI) of the outcome of the mediation effect did not contain zero, then the mediation effect would be significant at the 0.05 level. Table 2 shows the indirect effects and their associated 95% CIs. As shown in the table, the total indirect effect (i.e., the difference between total effect and direct effect) of stress through social support and resilience on life satisfaction was significant (β = −0.1293, *p* < 0.001). The single mediation of social support, the multiple serial mediations of social support and resilience, and the single mediation of resilience in the relationship between stress and life satisfaction were all significant in the tested model.

**Table 2:** Bootstrapping indirect effects and 95% confidence intervals(CI) for the final mediational model.

**Number**	**Model pathways**	**Point estimates β**	**95%CI**	
			**Lower**	**Upper**
1	Total Indirect Effect	−0.1293	−0.1992	−0.0738
2	Stress → SS → Life satisfaction	−0.0382	−0.0854	−0.0076
3	Stress → SS → Re → Life satisfaction	−0.0181	−0.0416	−0.0044
4	Stress → Re → Life satisfaction	−0.0576	−0.1064	−0.0163

*N = 426, SS = Social Support, Re = Resilience*.

## Discussion

In this study, we analyzed the mediating roles of social support and resilience in the relationship between stress and life satisfaction among individuals with substance use disorder in China. We hypothesized that stress is negatively related to life satisfaction, social support, and resilience. The correlational analyses indicated that our hypothesis is correct. Many previous studies have analyzed the relationships among stress, life satisfaction (66, 67), and resilience (38, 39). Furthermore, our correlational analyses showed that the relationship between stress and social support is negative. This result matches with those of the previous studies (68), which indicated that people who have substance disorder with low stress levels may maintain high social support.

Moreover, the mediating effects of social support and resilience on the relationship between stress and life satisfaction were significant among people with substance use disorder. Individuals with low stress levels can maintain higher social support than others, which enhances their resilience. All these advantages will enhance their life satisfaction levels. The results indicate that the life satisfaction of people with substance use disorder can be enhanced in many ways. Their stress level can be alleviated and their social support or resilience can be enhanced, thereby increasing their life satisfaction. Several previous studies have indicated that social support and resilience can decrease stress, and consequently, enhance life satisfaction. Hamama et al. (66) found that reducing the stress may enhance life satisfaction by increasing the social support from others. Shi et al. (45) found that resilience mediates the relationship between stress and life satisfaction.

From the final model of the present study, the path “stress → social support → resilience → life satisfaction” is significant. This path shows that individuals who have substance use disorder with low stress levels will receive more social support from others, which may increase their resilience, and consequently, their life satisfaction. Moreover, the research results indicate that social support and resilience are mediators between stress and life satisfaction. On the one hand, social support is a positive factor and predictor of increased life satisfaction; it can widen a person's social network, resist or relieve stress, and promote health (69, 70). Considerable research has identified the positive relationship between social support and life satisfaction (71). The results of the current study indicate that social support is a mediator between stress and life satisfaction. People who have substance use disorder with low stress levels may receive more social support, thereby increasing their life satisfaction. On the other hand, the mediating effect of resilience is consistent with the results of the previous studies (45). Research shows that chronic stress is positively related to reduced resilience, particularly in the face of loss (72). In addition, resilience is considered an important factor in the development of a person's life satisfaction (44, 73). On the basis of these results, inferring that resilience plays a mediating role in the relationship between stress and life satisfaction is reasonable. This study provides initial support for this hypothesis.

In summary, this study extended insights into the complex interactions among the stress, social support, resilience, and life satisfaction of Chinese individuals who have substance disorder. The important path from stress to social support to resilience to life satisfaction determines the internal mechanism between stress and life satisfaction. The results of the current study can provide valuable guidance in implementing psychological interventions to improve the life satisfaction of people with substance use disorder. Avoiding stress can be used as a preventive therapy to help such people to improve their life satisfaction. It can also be used as an active therapy to help them manipulate the impact of social support on their social relationships and mental resilience, thereby improving their life satisfaction.

However, the current study has several limitations. First, the data are completely dependent on face-to-face survey measures, which are prone to bias because the participants (i.e., people with substance use disorder) tend to provide responses with specific social needs. To reduce the impact of subjectivity, multiple assessment methods should be used for assessment. Second, the cross-sectional design of this study does not determine causality. In future studies, longitudinal and experimental methods can be used to analyze the relationships among stress, perceived social support, resilience, and life satisfaction. Third, the results of the current research are based on 2D measures of social support and resilience. In future studies, other facets of stress must be examined. Other possible mediating factors, such as loneliness, self-esteem, and happiness, must also be explored. Lastly, the sample in the current study was obtained from the population of individuals with substance use disorder. Thus, whether the current findings can be generalized to other population groups, such as the youth, males, females, and the elderly, requires further investigation.

## Ethics statement

This study was approved by the ethics committee of Nanjing Medical University, which fully considers safety and fairness principles. The research content will not pose any harm or danger to any group. The recruitment of the subjects will be entirely voluntary and informed consent will be obtained from the participants. The researchers exert maximum protection to guarantee the privacy of the subjects, the research contents, and the research results.

## Author contributions

There are a total of four authors are involved in this paper. CY and MX contributed equally to this work. MX is responsible for this paper, literature review and discussion part. CY is responsible for the whole framework and data processing as well as part of this paper. MH is responsible for the parts in this paper, the method and the polishing of the essay. YL is responsible for guiding the writing of this article.

### Conflict of interest statement

The authors declare that the research was conducted in the absence of any commercial or financial relationships that could be construed as a potential conflict of interest.
